# Sirtuin Family Members Selectively Regulate Autophagy in Osteosarcoma and Mesothelioma Cells in Response to Cellular Stress

**DOI:** 10.3389/fonc.2019.00949

**Published:** 2019-09-24

**Authors:** Richa Garva, Chutamas Thepmalee, Umpa Yasamut, Sangkab Sudsaward, Alice Guazzelli, Ramkumar Rajendran, Nopprarat Tongmuang, Sasiprapa Khunchai, Parisa Meysami, Thawornchai Limjindaporn, Pa-thai Yenchitsomanus, Luciano Mutti, Marija Krstic-Demonacos, Constantinos Demonacos

**Affiliations:** ^1^Faculty of Biology Medicine and Health, University of Manchester, Manchester, United Kingdom; ^2^Division of Biochemistry, School of Medical Sciences, University of Phayao, Phayao, Thailand; ^3^Division of Molecular Medicine, Research Department, Faculty of Medicine Siriraj Hospital, Mahidol University, Bangkok, Thailand; ^4^Division of Clinical Immunology, Department of Medical Technology, Faculty of Associated Medical Sciences, Chiang Mai University, Chiang Mai, Thailand; ^5^Department of Anatomy, Faculty of Medical Science, Naresuan University, Phitsanulok, Thailand; ^6^School of Environment and Life Sciences, University of Salford, Salford, United Kingdom; ^7^Faculty of Health and Medical Sciences, The University of Adelaide, Adelaide, SA, Australia; ^8^Department of Anatomy, Faculty of Medicine Siriraj Hospital, Mahidol University, Bangkok, Thailand; ^9^Center for Biotechnology, Sbarro Institute for Cancer Research and Molecular Medicine, College of Science and Technology, Temple University, Philadelphia, PA, United States

**Keywords:** autophagy, transcription, sirtuins, beclin-1, LC3, mesothelioma, osteosarcoma

## Abstract

The class III NAD^+^ dependent deacetylases-sirtuins (SIRTs) link transcriptional regulation to DNA damage response and reactive oxygen species generation thereby modulating a wide range of cellular signaling pathways. Here, the contribution of SIRT1, SIRT3, and SIRT5 in the regulation of cellular fate through autophagy was investigated under diverse types of stress. The effects of sirtuins' silencing on cell survival and autophagy was followed in human osteosarcoma and mesothelioma cells exposed to DNA damage and oxidative stress. Our results suggest that the mitochondrial sirtuins SIRT3 and 5 are pro-proliferative under certain cellular stress conditions and this effect correlates with their role as positive regulators of autophagy. SIRT1 has more complex role which is cell type specific and can affect autophagy in both positive and negative ways. The mitochondrial sirtuins (SIRT3 and SIRT5) affect both early and late stages of autophagy, whereas SIRT1 acts mostly at later stages of the autophagic process. Investigation of potential crosstalk between SIRT1, SIRT3, and SIRT5 revealed several feedback loops and a significant role of SIRT5 in regulating SIRT3 and SIRT1. Results presented here support the notion that sirtuin family members play important as well as differential roles in the regulation of autophagy in osteosarcoma vs. mesothelioma cells exposed to DNA damage and oxidative stress, and this can be exploited in increasing the response of cancer cells to chemotherapy.

## Introduction

Sirtuins are a family of seven proteins (SIRT1–7) of which some members exert histone deacetylase (HDAC) and/or ADP ribosyltransferase (ART) activity (1) or mediate other posttranslational modifications such as desuccinylation, demalonylation, and deglutarylation (2). The distinguishing characteristic of sirtuins is that in contrast to the members of the other HDAC classes (I, II, and IV) the class III members depend on the presence of increased levels of the oxidized nicotinamide adenine dinucleotide (NAD^+^) (3), which links their function to cellular energy pathways, and the regulation of oxidative stress (4, 5).

Sirtuin family members modulate transcription and the cellular redox state (6–8) and by doing so they orchestrate the outcome of a variety of cellular functions including autophagy, apoptosis, cell cycle control, response to DNA damage, and other signaling cascades (9–13). In particular, SIRT1 participates in nuclear DNA damage repair by regulating the PARP1 activity and in mitochondrial homeostasis through deacetylating PGC1α as well as participating in other pathways (14). The mitochondrial SIRT3 regulates nuclear-mitochondrial crosstalk in response to DNA damage, is involved in Ku70-dependent DNA damage repair (15), the generation of reactive oxygen species in mitochondria and in the mtDNA damage repair (16). Depending on the type of cancer SIRT5 has been reported to act as both oncogene (17) and tumor suppressor (18). The role of SIRT5 in DNA damage response is not well-studied but some literature suggests its potential function in ammonia-induced autophagy and mitophagy (19). SIRT5 promotes resistance to drugs inducing DNA damage in Non-Small Cell Lung Cancer (NSCLC), suggesting its potential as therapeutic target (20).

Sirtuins are important regulators of the evolutionarily conserved degradative autophagy process affecting this pathway in several different ways (21, 22). It has been reported that there is simultaneous regulation of SIRT1 and autophagy (23) and induction of SIRT1 by caloric restriction (CR) (24). Autophagy plays dual role as it both promotes cell survival (25, 26) and cell death (27), depending on the cell type, the type of stress, and the extent of damage. Some aspects of the role of SIRT1 in cell fate control in osteosaroma (28) and mesothelioma (29, 30) have been described as well as increased response to chemotherapy upon inhibition of autophagy in mesothelioma (31) but the role of the other sirtuin family members in autophagy and response to drug treatment in osteosarcoma and mesothelioma has not been clearly elucidated.

In the present study, the effects of different sirtuin family members on autophagy, the cell type or disease specificity of these effects, as well as the possibility to increase the therapeutic response to chemotherapy by targeting different sirtuins family members was investigated in osteosarcoma and mesothelioma cell lines. The results reported here suggest that in U2OS cells SIRT1 induced opposite pathways than SIRT3 and SIRT5, leading to cell death (SIRT1) or cell survival (SIRT3 and SIRT5) in response to DNA damage. In mesothelioma cell lines, SIRT3 and SIRT5 exerted pro-survival trends. These effects can in part be explained by the differential and selective control of autophagy by sirtuin family members and crosstalk between individual sirtuin family members.

## Materials and Methods

### Cell Lines, Cell Culture, and Constructs

U2OS, Mero-14, and REN cells were cultured in DMEM (Sigma Aldrich, UK) supplemented with 10% heat inactivated fetal calf serum (Gibco, UK) and 1% of penicillin and streptomycin 10,000 U/ml (Sigma Aldrich, UK) at 37°C in a humidified atmosphere containing 5% CO_2_. Wherever mentioned, cells were treated with 10 μM of the mitochondrial respiratory chain complex I inhibitor rotenone (32), the topoisomerase II inhibitor etoposide (33) (Sigma Aldrich, UK) or the SIRT1 and autophagy modulator resveratrol (Enzo Life Sciences, NY, USA) for 24 h.

The Flag-SIRT1 and SIRT3 expression constructs (34) were purchased from Addgene (Middlesex, UK) (35), and the SIRT5 was obtained from Dr. Chua (Stanford University School of Medicine, Stanford) (36). The polyfect transfection system (Qiagen, UK) was used to transfect cells according to the manufacturer's instructions.

### Sulforhodamine B (SRB) Assay

Transfection of siRNA against SIRT1, SIRT3, SIRT5, and non-targeting siRNA was carried out in 5 × 10^3^ cells/well in 96-well plates for 48 h. Transfected cells were treated with DMSO or the drugs, rotenone, etoposide, and resveratrol with various concentrations as indicated for 48 h. After treatment, cells were fixed with 10% trichloroacetic acid (TCA) for 1 h and then dried overnight at room temperature. Cells were stained with SRB dye for 15 min, washed twice with 1% acetic acid, and air dried for at least 1 h. The incorporated dye was dissolved in 10 mM Tris pH 8.8 solution and then plate was analyzed using a colorimetric microplate reader (Thermo Electron Multiskan Ascent Microplate Reader) (Akribis Scientific, Knutsford, UK).

### Immunoblotting and Antibodies

Cells were harvested in high salt lysis buffer (45 mM HEPES pH 7.5, 400 mM NaCl, 1 mM EDTA, 10% glycerol, 0.5% NP-40, 1 mM DTT, 1 mM PMSF, protease inhibitors including 1 μg/ml aprotinin, 1 μg/ml leupeptin, 1 μg/ml pepstatin, phosphatase inhibitors including 20 mM β-glycerophosphate, 5 mM sodium pyrophosphate, and 2 mM sodium orthovanadate) and equal amounts of protein were loaded and resolved by SDS-PAGE and Western blotting. The specific primary antibodies for β-Actin (Abcam), Beclin-1 (Cell Signaling), LC3 (Cell Signaling), SIRT1 (B-7, Santa Cruz Biotechnology), SIRT3 (C73E3, Cell Signaling), SIRT5 (D5E11, Cell Signaling), and anti-Flag M2 (Sigma Aldrich) were diluted 1:2,000 in 5% skimmed milk in Tris-buffered saline-Tween (TBS-T). Secondary antibodies were diluted 1:1,000 and incubated for 1 h. Blots were developed with ECL substrate according to manufacturer's instructions (Pierce, Thermo Scientific, USA).

### Quantitative RT-PCR

Quantitative RT-PCR analysis was carried out as described previously (37). Briefly, total RNA was extracted from cells using RNeasy plus mini kit (74134, Qiagen, USA) following manufacturer's instructions. The RNA was then reverse transcribed to cDNA (BIO-65042, Bioline cDNA synthesis kit) and used for qRT-PCR analysis using SYBR Green fluorescent probe. Specific primers for LC3 were used to perform qRT-PCR and analysis of the results was carried out using the OpticonMonitor 3.1 software. All values were normalized to the RPL-19 control. The primer sequences used in qRT-PCR reaction are provided in Supplementary Table 1.

### Small Interfering RNA

Cells were transfected with siRNA against SIRT1, SIRT3, SIRT5, and non-targeting siRNA was used as a control. siRNAs were transfected with DharmaFECT(# T-2001) according to the manufacturer's (GE Dharmacon) instructions. Cells were maintained in 10% FBS DMEM 48 h after transfection. The siRNA sequences targeting SIRT1, SIRT3, SIRT5, and the non-targeting scramble siRNAs are provided in the Supplementary Table 2.

### GFP-LC3 Punctate Assay

Cells were transfected with siRNA against SIRT1, SIRT3, SIRT5, non-targeting siRNA, and GFP-LC3 plasmid (Addgene, # 24920) using Lipofectamine 2000 reagent (Thermo Fisher Scientific) for 24 h. After that, 10 μM rotenone, 10 μM etoposide, and 50 μM resveratrol were used to treat the cells for 24 h. Cells were collected and analyzed using BD FACSVerse™ Flow Cytometer (BD Biosciences). The reduction of the GFP-LC3 mean fluorescence intensity is an indicator of autophagy activation.

### Flow Cytometric Analysis of Autophagy

FACS analysis and the monodansylcadaverine (MDC) that preferentially accumulates in autophagic vacuoles were used (38–41) to assess autophagy. U2OS cells were cultured for 24 h in 6-well plates. The cells were treated with rotenone and etoposide for 24 h. The collected cells were suspended in 0.05 mM autophagy vacuole specific dye MDC at 37°C for 10 min (42). For experiments where cells were transiently transfected, CD20 expressing vector was co-transfected with other plasmids and used as an indicator of transfection efficiency. CD20 expresses the B-lymphocyte antigen CD20. Exogenously introduced CD20 is delivered on cell surface which is tagged by APC-H7 conjugated CD20 IgG antibody. After dissociation cells were incubated with 1 ml of APC-H7 conjugated CD20 antibody (1 μg/ml, 1:1,000, BD Biosciences) in cell culture medium on a rotator at room temperature for 1 h. Population of cells that retained APC-H7 fluorescence emission were selected for MDC measurement. Then cells were analyzed with flow cytometer (Becton Dickinson). The fluorescent intensity of intracellular MDC reflected the number of autophagic cells.

### Statistical Analysis

Statistical analysis was performed using GraphPad software. Error bars represent standard error of means. One-way analysis of variance (ANOVA) and Tukey's multiple comparisons post-test were used for the analysis of the data. *p* values lower or equal to 0.05 were considered statistically significant.

## Results

### Effect of SIRT Family Members on Drug Cytotoxicity

Given the role of the sirtuin family members in regulating vital cellular functions the contribution of individual sirtuin family members to cell survival was investigated. The effects of silencing of the nuclear SIRT1 vs. the mitochondrial SIRT3 and SIRT5 family members on cell viability were analyzed in the human osteosarcoma (U2OS) and mesothelioma (Mero-14 and REN) cell lines. Cells were treated with the mitochondrial respiratory chain complex I inhibitor rotenone (32) to test effects of oxidative stress, the topoisomerase II inhibitor etoposide (33) to analyse effects of DNA damage and the sirtuins activator and autophagy modulator resveratrol (43).

**Figure 1:**
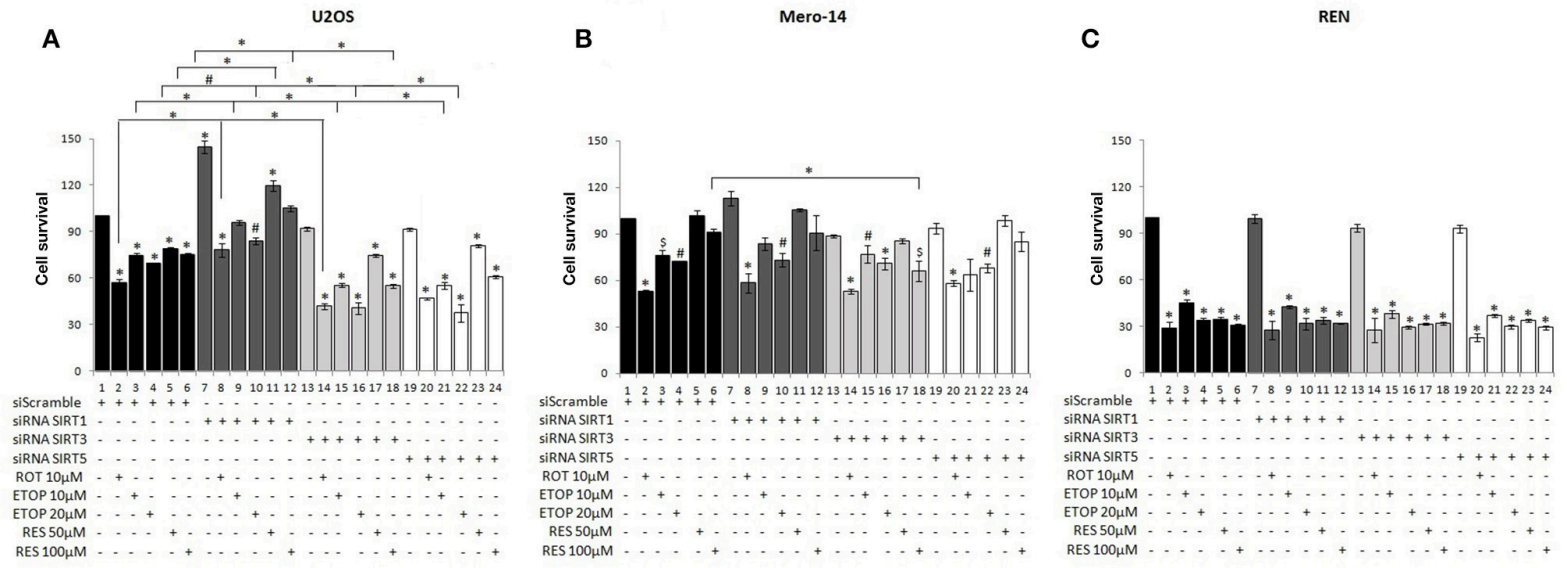
Effect of silencing of sirtuin family members on cancer cell viability under diverse types of cellular stress. SRB assay was used to assess cell viability of **(A)** U2OS, **(B)** Mero-14, and **(C)** REN cells in which SIRT1 (7–12), SIRT3 (13–18), or SIRT5 (19–24) had been transfected to silence the respective sirtuin family member as indicated. Cells were treated with rotenone, etoposide, and resveratrol for 48 h with the indicated concentrations. Bars 1–6 represent cell viability of cells transfected with siRNA scramble. Error bars represent standard error of means of three or more independent experiments. One-way analysis of variance (ANOVA) and Tukey's multiple comparisons post-test were used for the analysis of the data. *p* < 0.05 was considered to be statistically significant. *p* < 0.001 is indicated with (*), *p* < 0.01 with (#), and *p* < 0.05 with ($).

In scramble siRNA transfected U2OS and REN cells, all drug treatments decreased cell viability, whereas in scramble siRNA transfected Mero-14 cells only rotenone and etoposide and not resveratrol reduced cell survival (Figure 1). Silencing of the nuclear SIRT1 significantly increased cell viability in U2OS cells (Figure 1A, compare bars 1–6 to 7–12), whereas silencing of the mitochondrial SIRT3 and SIRT5 did not affect cell survival in the absence of treatment (Figure 1A, compare bar 1 to bars 13 and 19, respectively). SIRT1 silencing in U2OS cells treated with rotenone, etoposide, and resveratrol resulted in significant increase of cell viability compared to scramble transfected cells treated with the same drugs (Figure 1A, compare bars 2–6 to 8–12). On the contrary, silencing of the mitochondrial SIRT3 and SIRT5 in U2OS cells led to decreased cell viability upon etoposide treatment (Figure 1A, compare bars 3, 4 to 15, 16 and 21, 22 respectively). In addition, reduced cell viability was observed in resveratrol treated U2OS cells transfected with siRNA targeting SIRT3 (Figure 1A, compare bar 6 to 18).

To determine whether the observed effects of sirtuin family members on cell viability in U2OS cells were similar in other types of cancer, SRB assay was carried out in Mero-14 mesothelioma cells transfected either with scramble or siRNA targeting SIRT1, SIRT3, or SIRT5. Silencing of the nuclear SIRT1 and the mitochondrial SIRT3 and SIRT5 did not affect significantly cell viability in Mero-14 cells in the absence of any treatment (Figure 1B, compare bar 1 to 7, 13, and 19). SIRT1 or SIRT5 silencing in Mero-14 cells did not affect significantly cell viability in the presence of rotenone, etoposide or resveratrol (Figure 1B, compare bars 1–6 to 7–12 and 19–24). In contrast, significantly decreased cell viability was evident in Mero-14 cells transfected with siRNA targeting SIRT3 and treated with resveratrol (Figure 1B, compare bar 6 to 18).

The effects of silencing of sirtuin family members on cell viability were also tested in the REN cell line which differs from Mero-14 in terms of expression of crucial genes involved in cancer cell viability and response to cytotoxic drugs including p53 (44, 45). Substantial and statistically significant downregulation of cell viability was observed in REN cells treated with the studied drugs, however silencing of SIRT1, SIRT3, and SIRT5 did not exert significant effects (Figure 1C) although downward trend was observed in SIRT3 and SIRT5 silenced cells.

In summary, silencing of SIRT1 led to increased U2OS cell viability in all conditions tested and SIRT5 silencing resulted in decreased cell viability in etoposide treated U2OS cells. SIRT3 and SIRT5 exerted opposing to SIRT1 effects on cell viability of U2OS cells exposed to diverse types of cellular stress (pro-survival the SIRT3 and SIRT5 and pro-death the SIRT1). Reduced cell viability was also observed in mesothelioma cells treated with rotenone and etoposide, as well as in resveratrol treated Mero-14 cells in which SIRT3 had been silenced.

### Inter-family Regulation of SIRT Family Members

**Figure 2:**
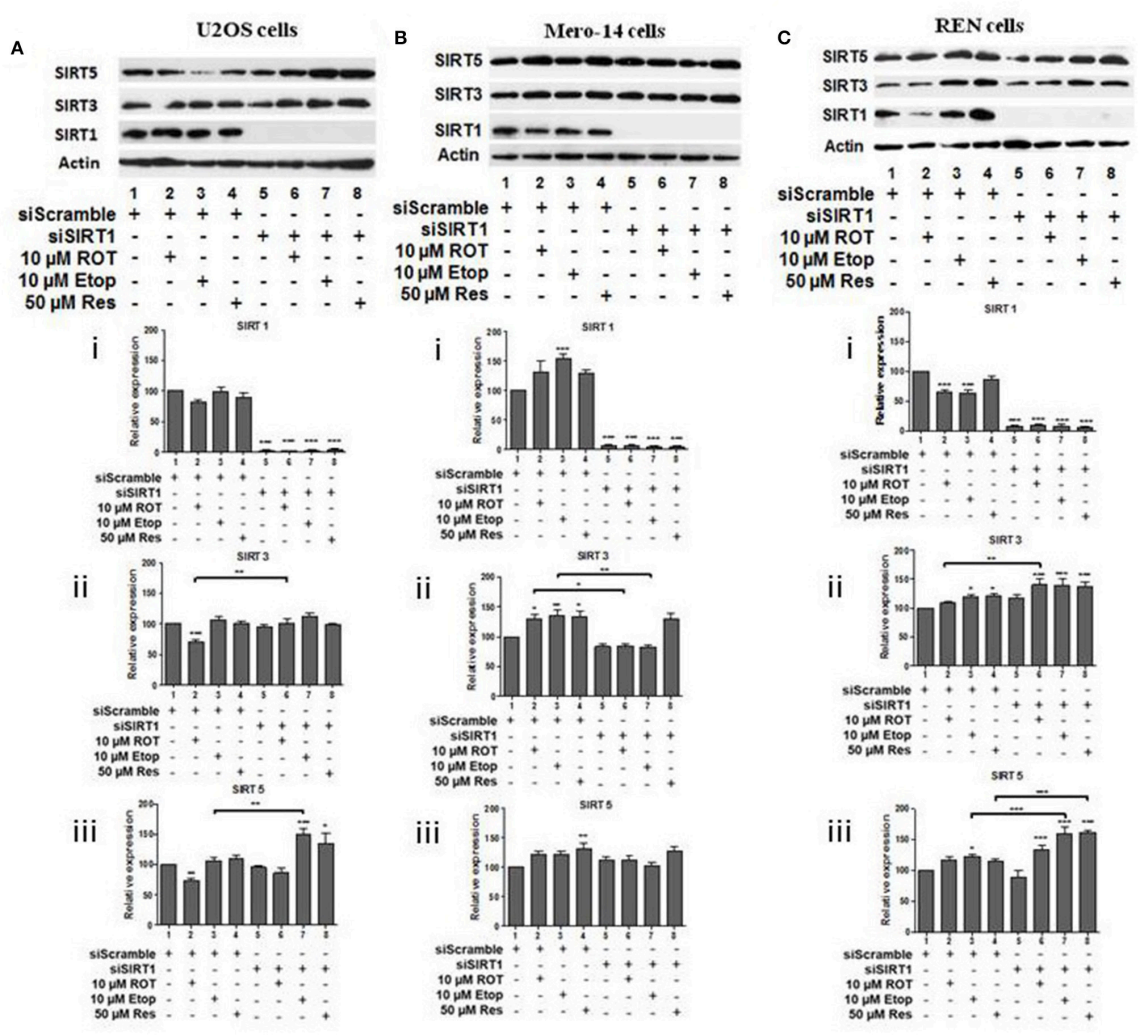
Effect of silencing of SIRT1 on SIRT3 and on SIRT5 protein levels. Protein levels of SIRT1, SIRT3, and SIRT5 were determined by SDS PAGE followed by Western blot. Silencing of SIRT1 gene expression in **(A)** U2OS, **(B)** Mero-14, and **(C)** REN cells. Protein levels were analyzed using antibodies specific for the SIRT1, SIRT3, or SIRT5. Actin was used as a loading control. Representative western blot is shown. Image J was used for quantification. Values are normalized to actin and then to control. Error bars represent standard error of the mean of three independent experiments. One-way analysis of variance (ANOVA) and Tukey's multiple comparisons post-test were used for the analysis of data. *p* < 0.05 was considered to be statistically significant. *p* < 0.001 is indicated with (***), *p* < 0.01 with (**), and *p* < 0.05 with (*).

**Figure 3:**
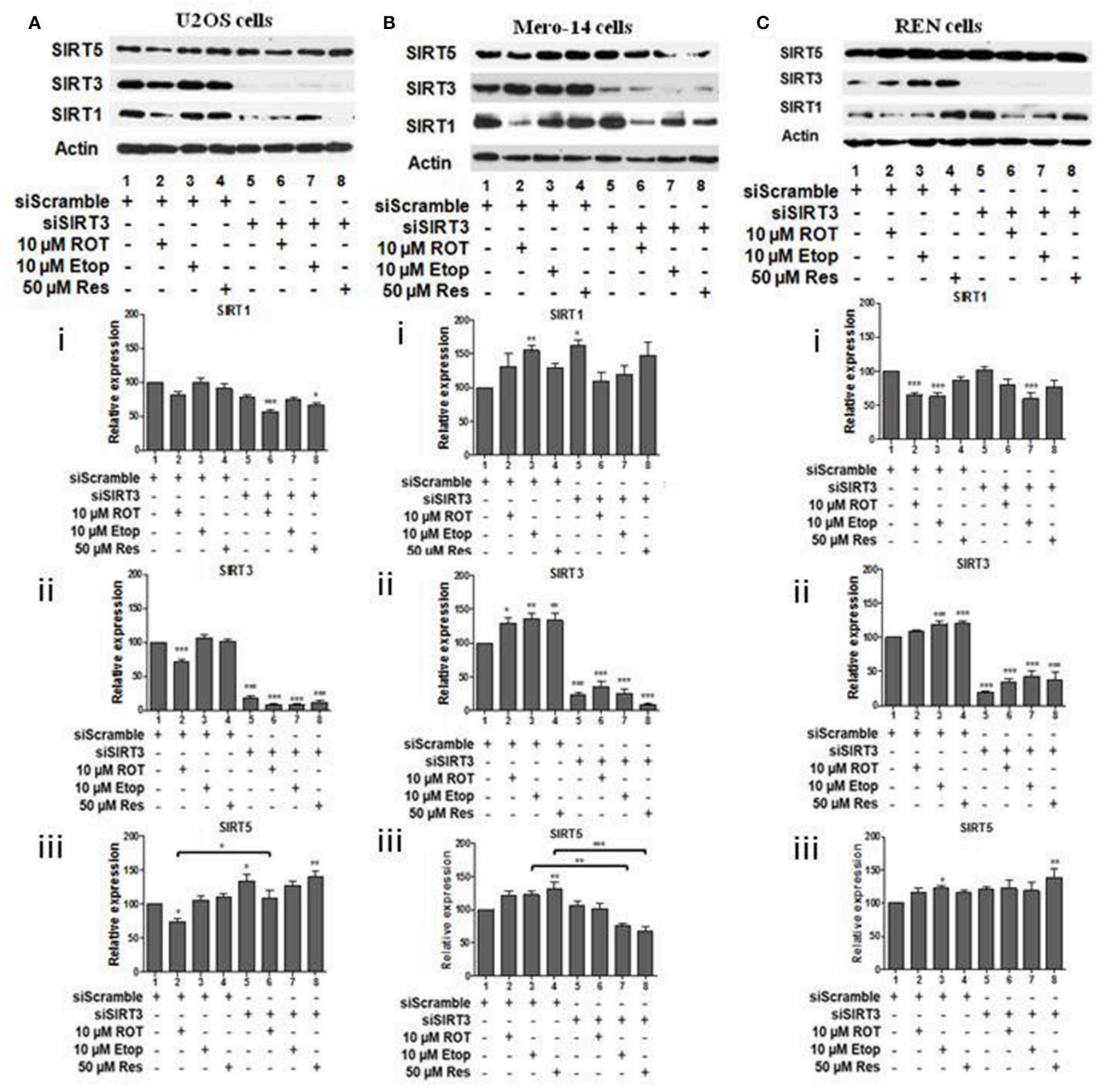
Effect of silencing of SIRT3 on SIRT1 and on SIRT5 protein levels. Protein levels of SIRT1, SIRT3, and SIRT5 were determined by SDS PAGE followed by Western blot. Silencing of SIRT3 gene expression in **(A)** U2OS, **(B)** Mero-14, and **(C)** REN cells. Protein levels were analyzed using antibodies specific for the SIRT1, SIRT3, or SIRT5. Actin was used as a loading control. Representative western blot is shown. Image J was used for quantification. Values are normalized to actin and then to control. Error bars represent standard error of the mean of three independent experiments. One-way analysis of variance (ANOVA) and Tukey's multiple comparisons post-test were used for the analysis of data. *p* < 0.05 was considered to be statistically significant. *p* < 0.001 is indicated with (***), *p* < 0.01 with (**), and *p* < 0.05 with (*).

**Figure 4:**
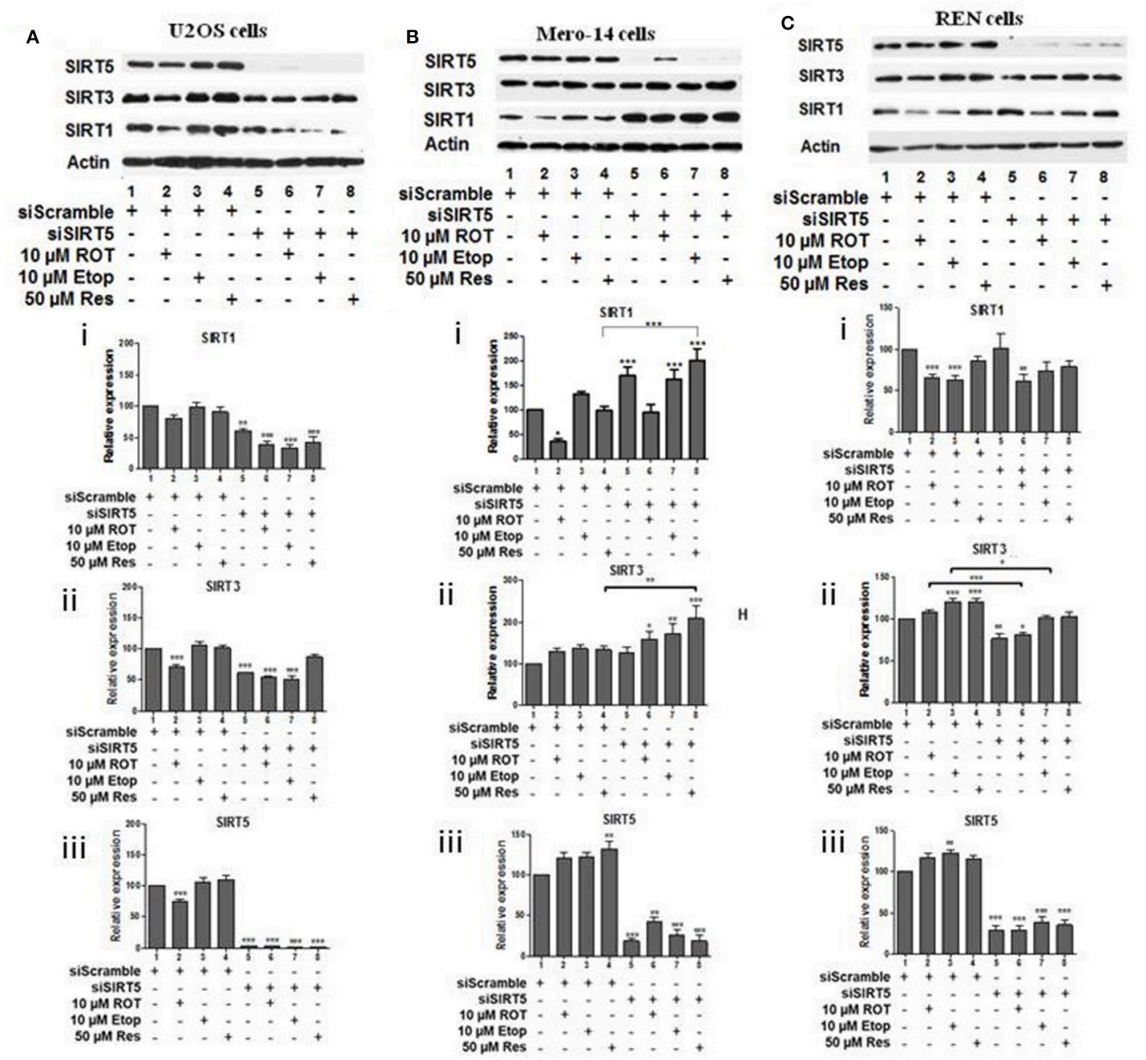
Effect of silencing of SIRT5 on SIRT1 and on SIRT3 protein levels. Protein levels of SIRT1, SIRT3, and SIRT5 were determined by SDS PAGE followed by Western blot. Silencing of SIRT5 gene expression in **(A)** U2OS, **(B)** Mero-14, and **(C)** REN cells. Protein levels were analyzed using antibodies specific for the SIRT1, SIRT3, or SIRT5. Actin was used as a loading control. Representative Western blot is shown. Image J was used for quantification. Values are normalized to actin and then to control. Error bars represent standard error of the mean of three independent experiments. One-way analysis of variance (ANOVA) and Tukey's multiple comparisons post-test were used for the analysis of data. *p* < 0.05 was considered to be statistically significant. *p* < 0.001 is indicated with (***), *p* < 0.01 with (**), and *p* < 0.05 with (*).

To investigate potential interdependency between individual family members, the protein levels of SIRT1, SIRT3, and SIRT5 were determined in U2OS, Mero-14, and REN cells in which each one of these sirtuin family members had been silenced. In U2OS cells, SIRT1 silencing increased SIRT3 protein levels in rotenone treated cells (Figure 2Aii, compare bars 2 and 6) and SIRT5 protein levels in etoposide treated cells (Figure 2Aiii, compare bar 3 to 7). In Mero-14 cells, SIRT1 silencing decreased SIRT3 protein levels in rotenone and etoposide treated cells (Figure 2Bii, compare bars 2 and 3 with bars 6 and 7). In REN cells, SIRT1 silencing increased SIRT3 protein levels in rotenone treated cells (Figure 2Cii, compare bar 2 to 6) and SIRT5 protein levels in etoposide and resveratrol treated cells (Figure 2Ciii, compare bars 3 and 4 to 7 and 8). In U2OS cells, SIRT3 silencing increased SIRT5 protein levels in untreated and rotenone treated cells (Figure 3Aiii, compare bars 1 and 2 to 5 and 6). In Mero-14 cells, SIRT3 silencing increased SIRT1 protein levels in untreated cells (Figure 3Bi, compare bar 1 to 5) and decreased SIRT5 protein levels in etoposide and resveratrol treated cells (Figure 3Biii, compare bars 3 and 4 to 7 and 8). Silencing of SIRT3 had no major effect in REN cells (Figure 3C). In U2OS cells, SIRT5 silencing resulted in downregulation of SIRT1 and SIRT3 protein levels (Figures 4Ai,ii, compare bars 1 to 5). In Mero-14 cells, SIRT5 silencing increased SIRT1 levels in the untreated and resveratrol treated cells and SIRT3 protein levels in resveratrol treated cells (Figures 4Bi,ii, compare bars 1 to 5 and 4 to 8, respectively). In REN cells, SIRT5 silencing decreased SIRT3 protein levels in untreated, rotenone, and etoposide treated cells (Figure 4Cii, compare bars 1, 2, and 3 to 5, 6, and 7).

In summary, complex interactions between SIRT1, SIRT3, and SIRT5 were identified, that changed upon oxidative stress and DNA damage. The majority of the feedback loops originated from SIRT5.

### Effect of SIRT Family Members on Beclin-1 and LC3 Autophagy Markers

**Figure 5:**
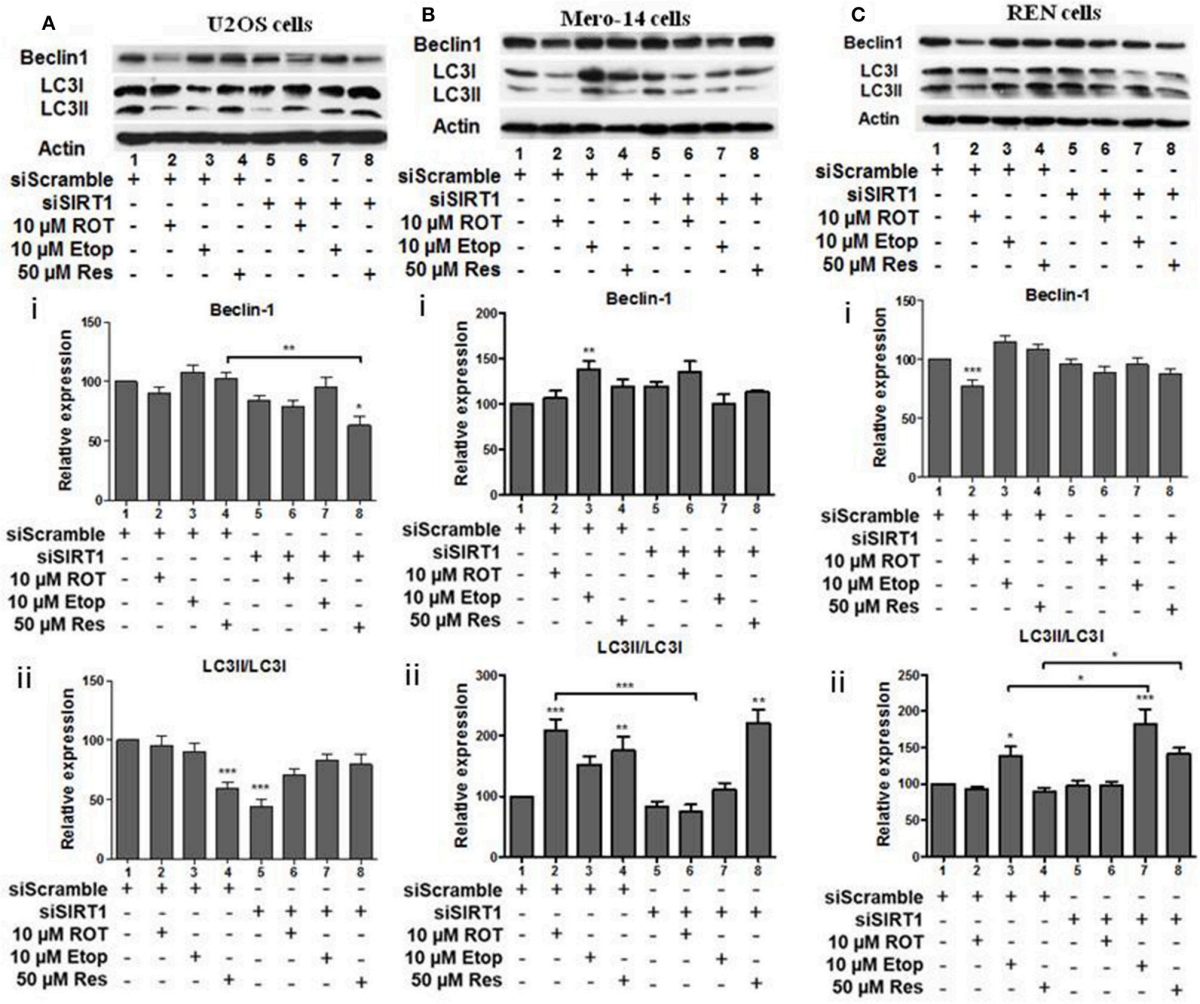
The effect of SIRT1 silencing on LC3 and Beclin-1 protein levels. **(A)** U2OS, **(B)** Mero-14, or **(C)** REN cells were transfected with either scrambled siRNA or SIRT1 siRNA and treated with rotenone, etoposide, or resveratrol or left untreated as indicated. Protein levels were analyzed using antibodies specific for the LC3 and Beclin-1 autophagy markers. Actin was used as a loading control and LC3II/LC3I ratio was calculated. Representative Western blot is shown. Image J was used for quantification. Values are normalized to actin and then to control. Error bars represent standard error of the mean of three independent experiments. One-way analysis of variance (ANOVA) and Tukey's multiple comparisons post-test were used for the analysis of data. *p* < 0.05 was considered to be statistically significant. *p* < 0.001 is indicated with (***), *p* < 0.01 with (**), and *p* < 0.05 with (*).

In the next set of experiments, effects of sirtuin family members on autophagy markers beclin-1 and LC3 were followed in U2OS human osteosarcoma cells, Mero-14 and REN mesothelioma cells. U2OS cells were incubated in HBSS as positive control to mimic starvation and confirm beclin-1 and LC3II/LC3I increase under these conditions (Supplementary Figure 1). SIRT1 silencing resulted in the downregulation of beclin-1 protein levels in U2OS cells treated with resveratrol (Figure 5Ai, compare bars 4 and 8). Down regulation of LC3II/LC3I ratio was observed in untreated U2OS cells in which SIRT1 had been silenced (Figure 5Aii, compare bar 1 to 5). In SIRT1 silenced Mero-14 cells, the LC3II/LC3I ratio was downregulated in rotenone treated cells (Figure 5Bii, compare bar 2 to 6). In REN cells carrying silenced SIRT1, the LC3II/LC3I ratio was upregulated in etoposide and resveratrol treated cells (Figure 5Cii, compare bars 3 and 4 to 7 and 8).

**Figure 6:**
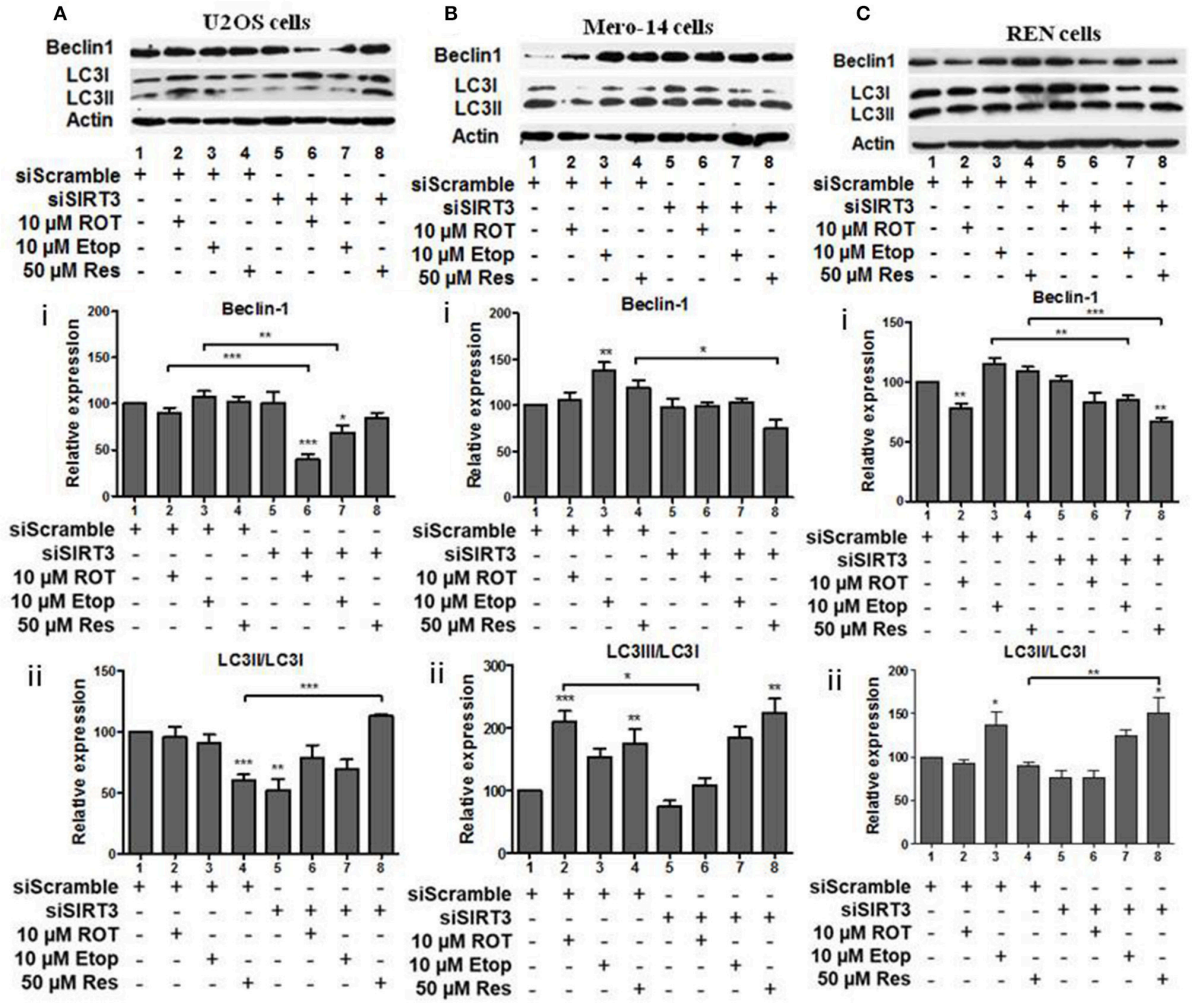
The effect of SIRT3 silencing on LC3 and Beclin-1 protein levels. **(A)** U2OS, **(B)** Mero-14, or **(C)** REN cells were transfected with either scrambled siRNA or SIRT3 siRNA and treated with rotenone, etoposide, or resveratrol or left untreated as indicated. Protein levels were analyzed using antibodies specific for the LC3 and Beclin-1 autophagy markers. Actin was used as a loading control and LC3II/LC3I ratio was calculated. Representative Western blot is shown. Image J was used for quantification. Values are normalized to actin and then to control. Error bars represent standard error of the mean of three independent experiments. One-way analysis of variance (ANOVA) and Tukey's multiple comparisons post-test were used for the analysis of data. *p* < 0.05 was considered to be statistically significant. *p* < 0.001 is indicated with (***), *p* < 0.01 with (**), and *p* < 0.05 with (*).

SIRT3 silencing led to downregulation of beclin-1 in U2OS cells treated with rotenone and etoposide (Figure 6Ai, compare bars 2 and 3 to 6 and 7). SIRT3 silencing down regulated the LC3II/LC3I ratio in untreated U2OS cells (Figure 6Aii, compare bar 1 to 5), whereas upregulation of this ratio was observed in the resveratrol treated SIRT3 silenced cells (Figure 6Aii, compare bar 4 to 8). In Mero-14 cells, in which SIRT3 had been silenced beclin-1 protein levels were downregulated in resveratrol treated cells (Figure 6Bi, compare bar 4 to 8). In Mero-14 cells bearing silenced SIRT3, the LC3II/LC3I ratio was downregulated in rotenone treated cells (Figure 6Bii, compare bar 2 to 6). In REN cells carrying silenced SIRT3 beclin-1 protein levels were downregulated in etoposide and resveratrol treated cells (Figure 6Ci, compare bars 3 and 4 to 7 and 8). In SIRT3 silenced REN cells, the LC3II/LC3I ratio was upregulated in the presence of resveratrol (Figure 6Cii, compare bar 4 to 8).

**Figure 7:**
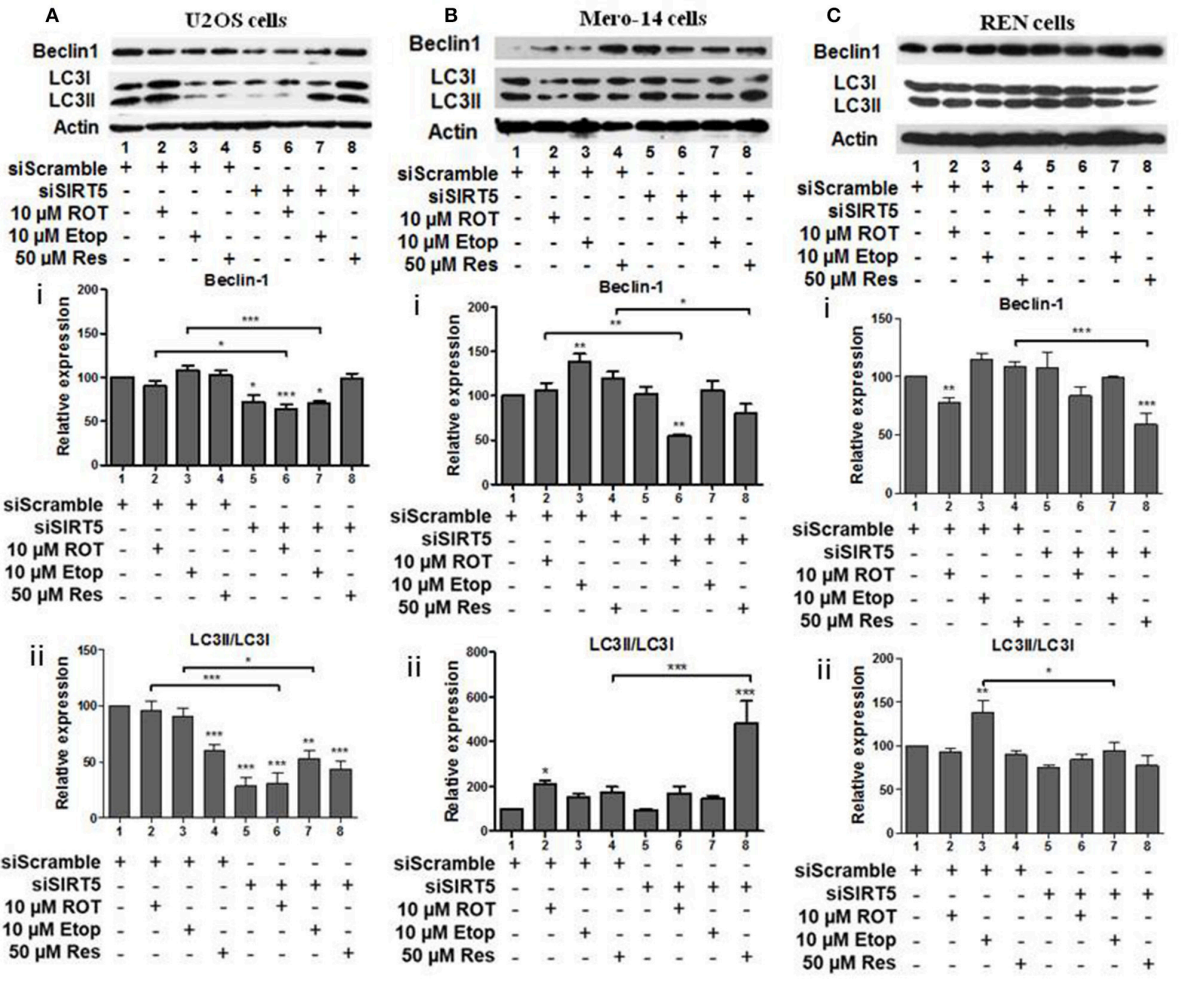
The effect of SIRT5 silencing on LC3 and Beclin-1 protein levels. **(A)** U2OS, **(B)** Mero-14, or **(C)** REN cells were transfected with either scrambled siRNA or SIRT-5 siRNA and treated with rotenone, etoposide, or resveratrol or left untreated as indicated. Protein levels were analyzed using antibodies specific for the LC3 and Beclin-1 autophagy markers. Actin was used as a loading control and LC3II/LC3I ratio was calculated. Representative western blot is shown. Image J was used for quantification. Values are normalized to actin and then to control. Error bars represent standard error of the mean of three independent experiments. One-way analysis of variance (ANOVA) and Tukey's multiple comparisons post-test were used for the analysis of data. *p* < 0.05 was considered to be statistically significant. *p* < 0.001 is indicated with (***), *p* < 0.01 with (**), and *p* < 0.05 with (*).

Beclin-1 protein levels and LC3II/LC3I ratio were downregulated in SIRT5 silenced untreated, rotenone, or etoposide treated U2OS cells (Figures 7Ai,ii, compare bars 1, 2, and 3 to 5, 6, and 7, respectively). In SIRT5 silenced Mero-14 cells, beclin-1 protein levels were downregulated upon rotenone and resveratrol treatment (Figure 7Bi, compare bars 2 and 4 to 6 and 8, respectively). LC3II/LC3I ratio was upregulated in resveratrol treated Mero-14 cells in which SIRT5 had been silenced (Figure 7Bii, compare bars 4 and 8). In REN cells, SIRT5 silencing downregulated beclin-1 protein levels in resveratrol treated cells (Figure 7Ci, compare bar 4 to 8). LC3II/LC3I ratio was downregulated in etoposide treated REN cells lacking SIRT5 expression (Figure 7Cii, compare bar 3 to 7).

In summary, in most cases, where significant effects were detected, SIRT1, SIRT3, and SIRT5 had positive effects on beclin-1 and LC3II/LC3I ratio, except in REN etoposide treated cells, where SIRT1 had negative effect on LC3II/LC3I ratio.

To explore whether the observed outcome of LC3II/LC3I ratio was a result of sirtuins mediated effects on transcriptional regulation of LC3, U2OS cells were transfected with SIRT1 or SIRT5 expression plasmids and total LC3 protein levels were followed. Ectopically expressed SIRT1 changed marginally but significantly the total LC3 protein and mRNA levels compared to pcDNA3 transfected cells (Supplementary Figures 2A–C, compare lane 1 to 2). SIRT5 overexpression modestly increased the total LC3 protein levels whereas LC3 mRNA levels did not change significantly (Supplementary Figures 2D–F, compare bar 1 to 2). These data provide evidence that SIRT1 and SIRT5 induce autophagy by inducing LC3 gene expression in U2OS cells.

### Effect of SIRT Family Members on Cellular Autophagy

**Figure 8:**
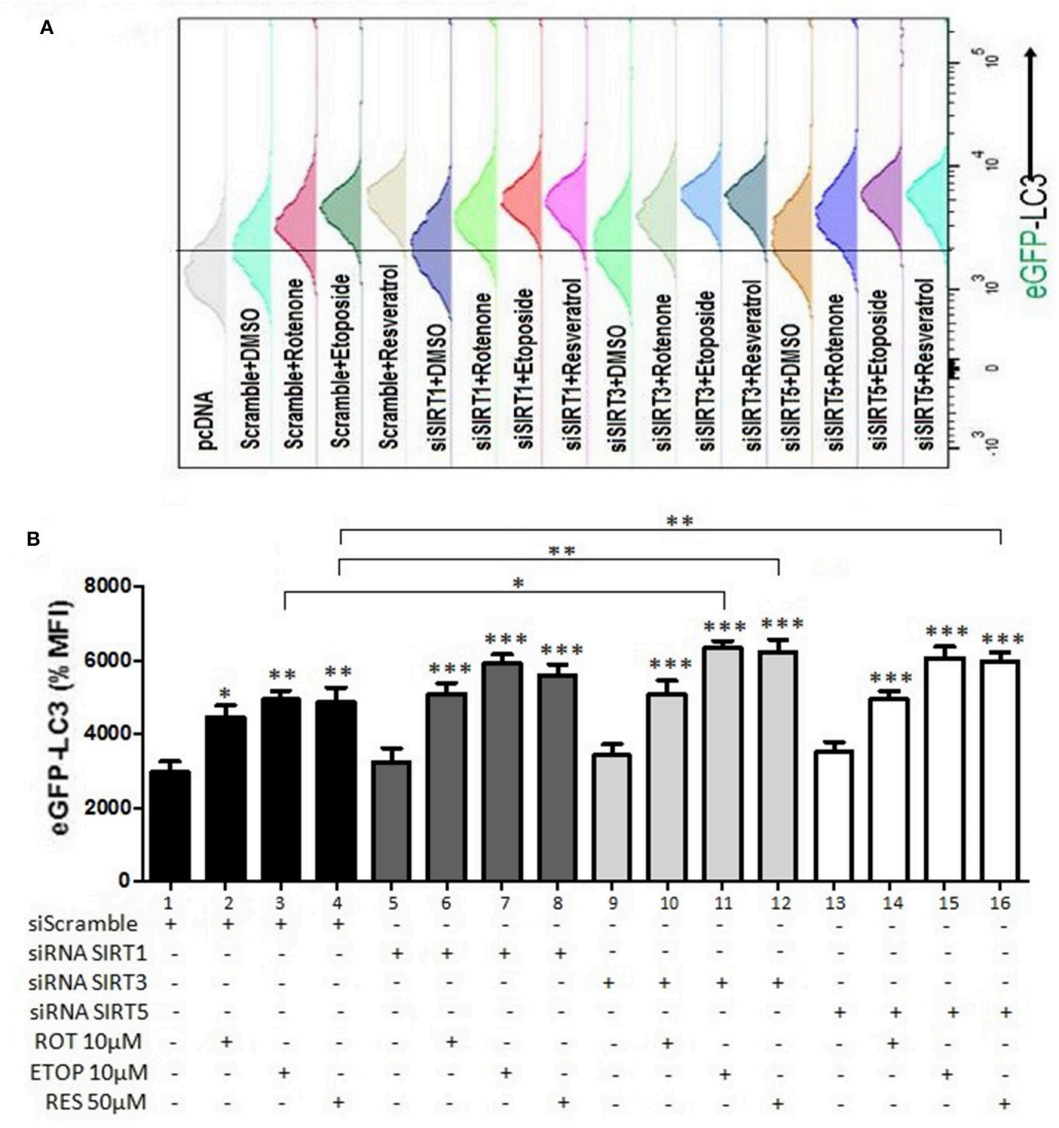
Regulation of autophagy by SIRT family members (GFP-LC3 punctate staining). **(A)** Histogram indicating the GFP-LC3 levels detected and quantified in U2OS cells transiently transfected with GFP-LC3 and a plasmid expressing the CD20 surface marker together with either scramble or siRNA targeting SIRT1, SIRT3, or SIRT5 and treated with rotenone, etoposide, or resveratrol as indicated. **(B)** Diagram indicating the analysis of the relative GFP-LC3 levels detected in U2OS cells transiently transfected with GFP-LC3 and a plasmid expressing the CD20 surface marker together with either scramble (1–4) or siRNA targeting SIRT1 (5–8), SIRT3 (9–12), or SIRT5 (13–16). The values obtained using flow cytometry in different conditions were normalized to those obtained from cells transfected with scramble and treated with DMSO which were arbitrarily set to 100. *p* < 0.001 is indicated with (***), *p* < 0.01 with (**), and *p* < 0.05 with (*).

Effects of sirtuin family members on autophagy markers beclin-1 and LC3 documented above indicated that this family plays an important role in autophagy control. To substantiate these findings, the LC3 punctate analysis was employed using flow cytometry (Figure 8). In this assay activation of autophagy should result in a decreased GFP-LC3 signal. Silencing of SIRT1, SIRT3, or SIRT5 in the absence of drug treatment did not affect autophagy (Figure 8B, compare bar 1 to 5, 9, and 13). Drug treatment had selective effect on autophagy in the presence and absence of individual sirtuins. Rotenone, etoposide, and resveratrol decreased autophagy in scramble siRNA transfected, as was evident by the increased LC3-GFP signal (Figure 8B, compare bar 1 to 2, 3 and 4). When SIRT1 was silenced, drug treatment did not affect autophagy substantially compared to the respective scramble control (Figure 8B, compare bars 2, 3, and 4 to 6, 7, and 8, respectively). SIRT3 silencing led to inhibition of autophagy in U2OS cells treated with etoposide or resveratrol (Figure 8B, compare bars 3 and 4 to 11 and 12), whereas SIRT5 silencing resulted in inhibition of autophagy in resveratrol treated U2OS cells (Figure 8B, compare bar 4 to 16). These results were confirmed by an additional assay assessing autophagy (MDC) (Supplementary Figure 3). Overexpression of sirtuins 3 and 5 in U2OS cells increased autophagy in both the absence and presence of rotenone and etoposide. Results shown in Figure 8 suggest that the individual sirtuins differentially modified drug effect on the autophagy process. SIRT1 had marginal effect on drug mediated control of autophagy, SIRT3 potentiated the effects of etoposide and resveratrol, whereas SIRT5 stimulated the effect of resveratrol on autophagy.

## Discussion

Autophagy is a degradative cellular machinery that eliminates damaged proteins and organelles thus serving as an important housekeeping pathway (46). One of the mechanisms modifying autophagy entails individual sirtuin family members. Reports investigating the role of SIRT1 as regulator of autophagy and response to cellular stress suggest that its effects are mainly mediated through its deacetylase activity (24) and can be both positive and negative (21, 22). SIRT1 knockout mouse embryonic fibroblasts exhibit decreased autophagy which coincides with increased levels of acetylated Atg5, Atg7, and LC3 (9). In osteosarcoma patients high expression of SIRT1 is associated with poor prognosis suggesting that SIRT1 promotes autophagy (28). In mesothelioma and lung cancer cells pemetrexed induces ROS generation and SIRT1 function thereby inducing apoptosis (28). In addition, SIRT1 has been shown to play a role in mTOR, AKT, and ER beta signaling in mesothelioma (29, 30). Although, the role of SIRT3 and SIRT5 in the control of cell fate has not been well-understood it has been shown that SIRT3 knockout mouse embryonic fibroblast cells exhibit increased autophagy in response to starvation in an LC3 independent manner (47). In addition, SIRT3 positively affects autophagy in neuronal cells and macrophages and exerts negative effect in hepatocytes (48, 49). SIRT5 is a key modulator of the response to starvation, metabolic homeostasis and cellular survival (50) promoting autophagy in colorectal cancer and its overexpression in this cancer is associated with poor survival (51). In breast cancer and mouse myoblasts cells SIRT5 silencing increases ammonia-induced autophagy through control of glutamine metabolism and mitophagy (19). Taken together these observations suggest that the relative contribution of sirtuin family members to autophagy is executed by distinct signaling networks which may depend on their enzymatic activity as deacetylases, cellular stress signals, and cell type (4). In this study the role of the sirtuins family members SIRT1, SIRT3, and SIRT5 in the regulation of autophagy was analyzed in the human osteosarcoma U2OS, and mesothelioma Mero-14 and REN cells treated with the inhibitor of the complex I of the mitochondrial respiratory chain rotenone, the topoisomerase II inhibitor etoposide widely used in the clinic, and the sirtuins activator and autophagy modulator resveratrol.

SIRT1 displayed cell type specific effects as it exerted pro-death activities in osteosarcoma but not in mesothelioma cells (Figure 1) possibly attributed to selective modulation of beclin-1 protein levels in U2OS cells (Figure 5). SIRT1 silencing in U2OS cells led to pro-survival outcome whereas silencing of this sirtuin family member in Mero-14 and REN cells did not have significant effect. The existence of differential micro-environmental conditions in osteosarcoma vs. mesothelioma cells that diversely affect the sirtuin family members mediated autophagy could be a potential explanation for these results. SIRT3 and SIRT5 exhibited pro-survival effects in U2OS cells, whereas in mesothelioma cell lines SIRT3 and SIRT5 exerted pro-survival trends (Figures 1A–C). These results could explain published observations indicating no significant improvement in the osteosarcoma patients treated with combination of chemotherapeutics including ifosfamide and etoposide, which have demonstrated activity in osteosarcoma in earlier studies (52–54). Conflicting roles of SIRT1 have been reported in osteosarcoma. High expression of SIRT1 was linked to high risk osteosarcoma patients (55), however, SIRT1 expression has also been reported to be down-regulated in metastatic osteosarcoma. The selective regulation on beclin-1 protein levels exerted by resveratrol only in mesothelioma and not in osteosarcoma cells is possibly due to the fact that NAD^+^/NADH ratio and ROS generation in the two cell types upon resveratrol treatment are regulated by different pathways (56).

Analysis of sirtuin family members crosstalk revealed that SIRT1 has negative effect on SIRT3 and SIRT5 protein levels in bone cells (Figure 2A) and this depends on the type of cellular stress, whereas positive effects were recorded in Mero-14 mesothelioma cells (Figure 2B). In general interdependency between sirtuin family members indicated that SIRT5 plays the most important role in regulating SIRT1 and SIRT3 protein levels in U2OS, Mero-14, and REN cells (Figures 4A–C) and this needs to be considered when interpreting results of experiments in which individual sirtuin family members are silenced. Overexpression of SIRT1 and SIRT5 in U2OS cells showed marginal positive effects on total LC3 protein and mRNA levels implying that these sirtuin family members exert their effects on autophagy through transcriptional and non-transcriptional pathways. Recent studies have shown that the mitochondrial desuccinylase and demalonylase activities of SIRT5 regulate autophagy and mitophagy (50) and a small nuclear fraction of SIRT5 exist (17) that could be involved in the SIRT5 mediated transcriptional regulation of autophagic genes (34).

If the effects on beclin-1 are considered early and those on LC3II/LC3I ratio as late autophagy events our results suggest that the nuclear SIRT1 acts mostly at late stages of autophagy and its effects are cell type/signal specific and depending on the experimental conditions they can be both positive and negative in the regulation of autophagy and cell survival. The mitochondrial sirtuins SIRT3 and SIRT5, on the other side, affect both early and late stages of autophagy are pro-proliferative under certain cellular stress conditions and their effects correlate with their role as positive regulators of autophagy.

Results presented in this study may have therapeutic implications given that SIRT1, SIRT3, and SIRT5 exert distinct roles in the regulation of autophagy in several cancer cell lines and therefore selective targeting of these sirtuin family members could be the basis for development of new strategies to increase cell response to chemotherapy. However, other factors such as the contribution of the microenvironmental conditions in fine tuning the enzymatic activities of sirtuins and hence the autophagy process require further investigation.

## Data Availability Statement

The datasets generated for this study are available on request to the corresponding author.

## Author Contributions

RG and CT planned and performed experiments, analyzed the results, and prepared the draft of the manuscript. UY, AG, SS, RR, NT, SK, and PM planned and performed experiments, analyzed the results and prepared part of the manuscript. TL, PY, and LM analyzed the results and prepared part of the manuscript. MK-D and CD formed the hypothesis, supervised the research carried out, interpreted the results and prepared the manuscript.

### Conflict of Interest

The authors declare that the research was conducted in the absence of any commercial or financial relationships that could be construed as a potential conflict of interest.
