# Supplementary material for: Sirtuin Family Members Selectively Regulate Autophagy in Osteosarcoma and Mesothelioma Cells in Response to Cellular Stress
Source: Front Oncol. 2019 Sep 24;9:949. doi: 10.3389/fonc.2019.00949 (PMC6771295; doi:10.3389/fonc.2019.00949)
Supplement: Supplementary file 1 [file Data_Sheet_1.PDF]

Supplementary figure 1

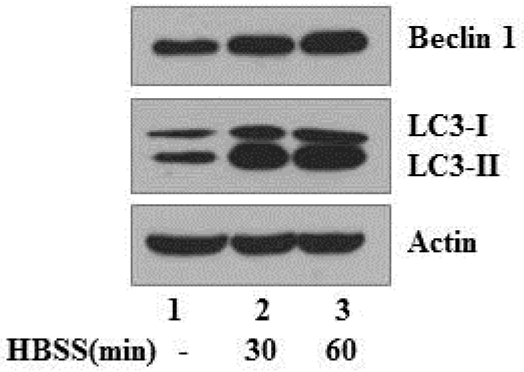

Induction of Beclin-1 and LC3 autophagy markers by nutrient starvation
Protein levels were analyzed by Western blotting, using antibodies specific for LC3, Beclin-1 and actin. Actin was used as a loading control. Nutrient starvation conditions (HBSS media) were used as positive control for induction of autophagy.

Supplementary figure 2

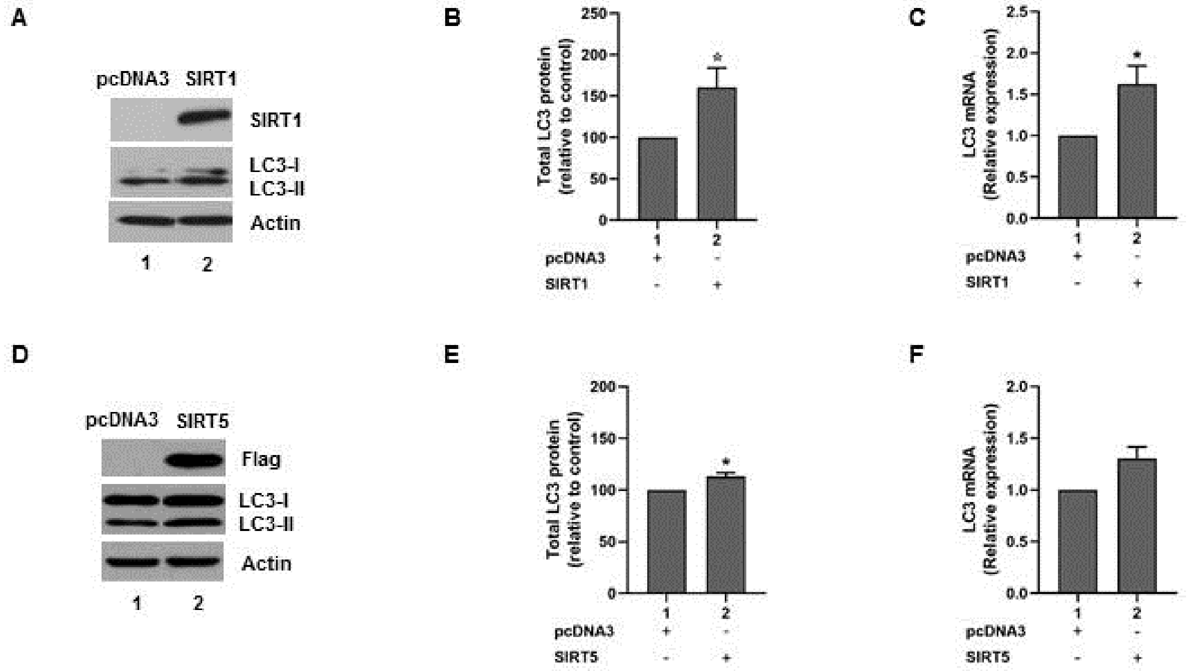

Effect of sirtuin family members' overexpression on LC3 protein and mRNA levels
(A) U2OS cells transfected with flag-tagged SIRT1 or (D) SIRT5 expression vector or pcDNA3 as control. Protein levels were analyzed using antibodies specific for LC3 and Flag tag to detect SIRT1 or SIRT5. Actin was used as a loading control. (B and E) Graphs show quantification of LC3 total protein levels normalized to actin levels. (C and F) LC3 mRNA levels were followed using qRT-PCR in U2OS cells overexpressing SIRT1 or SIRT5.

Supplementary Figure 3

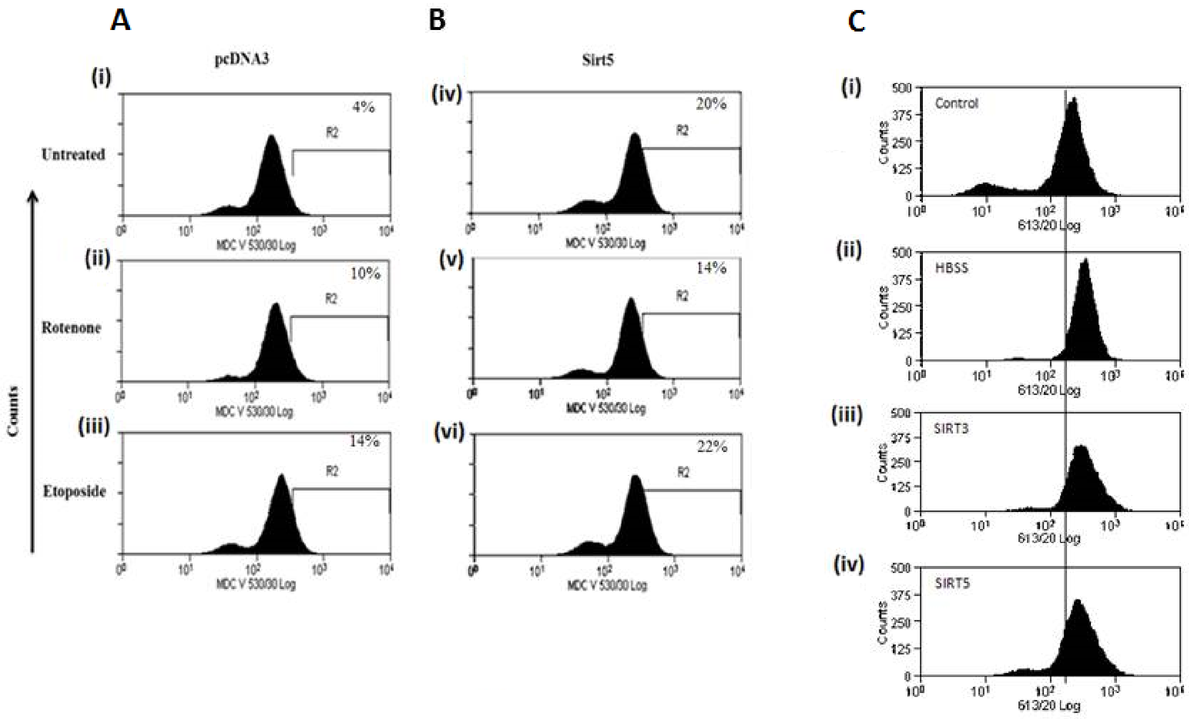

Regulation of autophagy by sirtuin family members (MDC assay)

(A) pcDNA3 and (B) SIRT5 transfected U2OS cells untreated (i) and (iv) or rotenone (ii) and (v) or etoposide (iii) and (vi) treated were stained with monodansylcadaverine and subjected to flow cytometric analysis for estimation of intracellular acidic vesicular organelle levels. (C) pcDNA3 (i) and (ii) or SIRT3 (iii) or SIRT5 (iv) transfected U2OS cells untreated (i), (iii) and (iv) or treated with HBSS media (ii) that induces autophagy serving as positive control were stained with monodansylcadaverine and subjected to flow cytometric analysis for estimation of intracellular acidic vesicular organelle levels.

Supplementary table 1

Oligonucleotides used for real-time quantitative PCR

**Primer**	**Sequence (5’-3’)**
LC3-F<br>LC3-R	TGTCCGACTTATTCGAGAGCAGCA<br>TGTGTCCGTTCACCAACAGGAAGA
RPL19-F<br>RPL19-R	ATGTATCACAGCCTGTACCTG<br>TTCTTGGTCTCTTCCTCCTTG

Supplementary table 2

Sequences of siRNA pools used in the study to silence the expression of SIRT1, SIRT3 or SIRT5 and the sequence of the non targeting siScramble used as a control.

**siRNA SIRT1 pool**
(1) GCAAAGGAGCAGAUUAGUA
(2) GCGAUUGGGUACCGAGAUA
(3) GGAUAGGUCCAUAUACUUU
(4) CCACCUGAGUUGGAUGAUA
**siRNA SIRT3 pool**
(1) UCACAUUACCUGCGUGUUU
(2) CCUGUGACUUUGCGCCUUA
(3) UUGAGAGAGUGUCGGGCAU
(4) GGACCAGACAAAUAGGAUG
**siRNA SIRT5 pool**
(1) UCGAUGAGCUGCACCGCAA
(2) GCUGAAUUUAACACGGAGA
(3) CCAGAUUGUCCCAAGUCGA
(4) CGUCGUGUGGUUUGGAGAA
**Non-Targeting siRNA pool**
(1) UAGCGACUAAACACAUCAA
(2) UAAGGCUAUGAAGAGAUAC
(3) AUGUAUUGGCCUGUAUUAG
(4) AUGAACGUGAAUUGCUCAA
